# A Multidisciplinary Study to Evaluate the Anti-quorum Sensing Ability of Phyto-compounds in *Ruellia patula* Jacq

**Published:** 2019

**Authors:** P Chemmugil, PTV Lakshmi, A Annamalai

**Affiliations:** 1. Centre for Bioinformatics, Pondicherry University, Pondicherry, India; 2. PG and Research Department of Botany, Arignar Anna Government College, Thiruvalluvar University, Villupuram, Tamilnadu, India

**Keywords:** Antibiotics, DNA binding domain, *Methicillin resistant Staphylococcus aureus*, Quorum sensing, *Staphylococcus aureus*

## Abstract

**Background:**

*Staphylococcus aureus* (*S. aureus*) causing numerous diseases in humans, have become resistant to antibiotics, hence, urging the need for alternative medicines.

**Methods:**

In this study, the Indian medicinal weed, *Ruellia patula (R. patula*) extracted and fractioned through column chromatography was subjected to antibacterial and anti-quorum sensing activity against *S. aureus* and Methicillin Resistant Staphylococcus aureus (MRSA).

**Results:**

The obtained results confirmed fraction F44 to have significant effect as antimicrobial and anti-biofilm agent against both the micro-organism. Therefore, few of such highly active fractions were chemical finger printed using GC-MS and the compounds identified were further docked with DNA binding (LytTR) domain of agrA, which revealed that compounds identified from fraction were interactive to the protein.

**Conclusion:**

*R. patula* is promising antimicrobial and anti-biofilm agent against *S. aureus* and MRSA.

## Introduction

New antibiotics are obtained either by chemical synthesis 1 or developed from natural sources. According to WHO, 80% of the world population use plant extracts as medicine due to its inexpensive and lower side effects 2. Strong antimicrobial fractions derived from plants are reported to inhibit bacterial growth by mechanisms like membrane disruption, metabolism damage, anti-biofilm, bacterial capsule prevention and virulence attenuation 1. In this study, *Ruellia patula (R. patula)* Jacq (syn. *Dipteracanthus patulus* Nees) popularly called as kiranthi nayagam, chilanthinayagam, kayap-pacchilai, Tutadi and sisodi belonging to Acanthaceae has been explored to identify its potential as the anti-biofilm agent. *R. patula* is distributed in tropical and subtropical regions of Africa, Arabia, Sri Lanka, Pakistan, Burma and India especially in Rajasthan, Haryana, Tamil Nadu, Western Ghats and Andhra Pradesh 3. It is an erect, woody plant growing up to 50 *cm* and has been used as a medicine for a variety of problems including gonorrhea, syphilis, sore eyes, renal infection, cough, scalds, stomach ache, kidney stones, tumors, rheumatic complaints, dental problems, itches, insect bites, paronychia, venereal diseases, for wound healing and as a cardiotonic and antiulcer agent. Juice of leaves acts as a sedative, roots as antipyretic agent, leaf paste as anti-inflammatory and flower and raw fruits as anti-diabetic agents 4. Thus, based on these medicinal values, preliminary investigations on the selection of the most suitable solvents for better recovery of compounds have been explored in this research 5.

In humans, gram positive bacteria *Staphylococcus aureus (S. aureus)* is responsible for a variety of infections including skin soft tissue infections 6, and infective endocarditis, urinary tract infection, acute septic arthritis and hospital acquired infections like device-related infection 7 especially under immune-suppression after organ transplantation and during chemotherapy 1 and bloodstream infections 8. Since *S. aureus* is able to produce biofilms which restrict diffusion of antibiotic and results 20 to 1000 times more drug concentration in comparison to planktonic bacteria 9, and is also antibiotic resistant 10 and thereby decrease the number of patients up to 30% 11, it is important to focus more on alternative strategies to develop new antibiotics 12. Since pathogenicity or adhesion and protection against host defenses of *S. aureus* strongly depends on the virulence factors such as production of exotoxin, enzymes and cell wall associated proteins, which are reported to be regulated by the accessory gene regulator (agr) of regulatory locus 13, and that its expression is reported to contribute to pathogenesis of biofilm associated infections in several models like murine subcutaneous abscesses, arthritis and rabbit endocarditis including invasion and apoptosis of epithelial cells 14, agr protein was identified as the target in the present study. Agr system contains *agrB*, *agrD*, *agrC*, and *agrA* genes, of which, *agrB* and *agrD* genes get involved in the production of Auto Inducer peptide (AI), which binds to agrC (Transmembrane protein) to induce transcription by activating the agrA (response regulator) 15. Thus, agrA up-regulates or represses the gene expression by binding the cognate DNA with DNA binding domain (also called LytTR domain), thereby regulates the expression of the gene involved in exotoxin synthesis 15 and controls the production of virulence factor in *S. aureus* 15,16.

Moreover, LytTR domain is reported to be present in all response regulators of prokaryotes (approx. 2.7%) and observed in all *S. aureus* strains identified including Methicillin Resistant Staphylococcus aureus (MRSA) along with certain other pathogens such as *Clostridium* sps. and *Listeria* sps. However absent in human 17 is of importance to us to be chosen as the target. Also, adequate number of subjects for the study is an important issue for conducting the research. Hence, it was hypothesized to evaluate the antimicrobial and quorum sensing activity of the fractions eluted through column chromatography against *S. aureus* and MRSA. Further, an attempt was made to identify the compounds present in those highly active fractions through GCMS and evaluate the lead molecules for interacting capacity with DNA binding (LytTR) domain of agrA.

## Materials and Methods

### Chemicals and instruments

Mueller Hinton broth and agar, Whatman filter paper No.1, analytical grade solvents such as Hexane, Chloroform, Ethyl acetate, Methanol, Dimethyl sulfoxide (DMSO) were all purchased from Himedia. Microplate analysis was done using Spectra Max M2e Microplate reader, Spinco Biotech, India. JEOLGCMATE II operating in EI mode at 70 eV (Agilent Technologies 6890N Network) was used for GC-MS analysis and spectrum was matched with NIST database. Softwares such as Marvin sketch, Autodock Vina, Autodock 4.2, Chimera, LigPlot+v.1.4, Graphp-ad prism were all used in this research.

### Fractionation through column chromatography

The whole plants of *R. patula* were shade dried, coarsely powdered (500 *g*) and extracted through cold maceration method with 2 *L* of 80% methanol at 40 °*C* for 72 *hr* in orbital shaker until the residues became pale colored. It was filtered using muslin cloth followed by whatman no.1 filter paper and condensed by rotary evaporator to obtain semisolid extract (48.76 *g*). Further, the semisolid extract was suspended in 500 *ml* of water and partitioned with 500 *ml* of Hexane (4.85 *g*). 20 *g* of concentrated water fraction was subjected to column chromatography while the remaining extracts were stored at 4 °*C*. The column was eluted through gradient solvent system, starting with 100% Hexane and Hexane combined with methanol in the ratio of 10:0; 9:1; 8:2 up to 5:5, followed by chloroform and ethyl acetate in the above mentioned combinations. Collected fractions were combined based on TLC profile, where approximately 60 different fractions were obtained and stored at 4 °*C* for further analysis 18.

### Maintenance of bacterial cultures

Clinical isolates of *S. aureus* and MRSA were collected from Department of Microbiology, Pondicherry University, Pondicherry and maintained in the Centre for Bioinformatics, Pondicherry University.

### Determination of antibacterial activity

In order to evaluate the antibacterial efficiency of the fractions, disk diffusion assay was followed 19 to determine the sensitivity of different fractions of *R. patula,* where both *S. aureus* and MRSA cultured on Mueller Hinton broth were incubated at 37±1 °*C* for overnight. Suspension with 1×10^6^ *CFU/ml* was swabed on the solid Mueller Hinton agar medium in petri dish. Disks with 20 *μl* of different fractions each were placed on the swabbed agar plates and incubated at 37±1°*C* for 24 *hr*. After incubation, the zones formed around the disks were measured and zone of inhibition was calculated. DMSO, which had no activity on bacteria, was used to dissolve fractions. The tests were conducted in triplicates.

### Determination of minimum inhibitory concentration (MIC)

Based on the results of the disk diffusion assay, those fractions which showed higher antibacterial activity were further evaluated for MIC by broth dilution method 20, where, two fold dilutions of fraction in the range of 100 *μg/ml* to 12.8 *mg/ml* were prepared by dissolving in DMSO. Wells were filled with 20 *μl* of overnight culture (1×106 *CFU/ml*) and 180 *μl* of Mueller Hinton broth followed by 100 *μl* of different concentrations of highly active fractions. DMSO alone was used as the negative control while, culture alone was maintained as the positive control and different concentrations of fractions with medium and without inoculums were used as blank and incubated for 18 *hr* at 37°*C* for absorption at 600 *nm*. Triplicates were maintained and MIC was determined by comparing the absorbance of fraction with control and expressed in *μg/ml*.

### Determination of synergistic activity

Based on the results of the antimicrobial activities of the eluted fractions, those which showed better activity were further examined to prove their action comparable to the antibiotic disks. Thus, disk diffusion method 21 was proceeded to determine synergic effect of active fractions (F25, F44 and F53) combined with commercial antibiotics like tetracycline (30 *μg/disk*), Chloramphenicol (30 *μg/disk*), and vancomycin (30 *μg/disk*). 20 *μl* of active fractions (1 *mg/ml*) were impregnated and placed on Mueller Hinton Agar plate to incubate for 18 *hr* at 37*°C*. The synergistic activity of fraction with the combination of drugs was determined by measuring the zone of inhibition.

### Performance of time killing assay

Based on the above observation, fraction 44 was further investigated for time killing assay 22. Next, 1 *ml* of each *S. aureus* and MRSA respectively and 1 *ml* of highly active fraction F44 (MIC determined concentration) were added and incubated at 37°*C* from which at intervals of 0, 1, 2, 6, 12 and 24 *hr*, 200 *μl* of fraction treated culture was harvested and serially diluted (10^−5^) to spread on Mueller Hinton agar to determine the killing rate by colony count. Untreated culture was kept as the control and the number of colonies counted was expressed as *CFU/ml*.

### Performance of anti-biofilm adhesion assay

Anti-biofilm adhesion activity of all 60 fractions against *S. aureus* and MRSA was carried out 7 in a 96 well plate that was pre coated with 200 *μl* of fractions (1 *mg/ml*) and was dissolved in DMSO and allowed to dry. 200 *μl* of DMSO alone coated was considered as negative control while, non-coated wells were left as positive controls. Approximately, 1×10^6^ *CFU/ml* of 20 *μl* of *S. aureus* and MRSA was inoculated into 180 *μl* of Mueller Hinton broth wells. 200 *μl* of Mueller Hinton broth added in to fraction pre-coated well was used as the blank and the experiment was done in triplicate. The plate was incubated for 24 *hr* without shaking at 37 °*C* and after 24 *hr*, the medium with loosely bound cells was removed and allowed to dry and washed three times with PBS. The remaining adhered cells were stained with 0.1% crystal violet for 30 *min*. Excess colorant was removed by rinsing with sterile distilled water and air dried. Further, 200 *μl* of 80% ethanol was added to the wells and OD was absorbed at 570 *nm*. The mean of the three replicates was calculated and the percentage of biofilm adhesion inhibition was calculated using the following formula.

$$\text{Inhibition}\;\%=\frac{\text{OD}\;\text{control}-\text{OD}\;\text{sample}}{\text{OD}\;\text{control}}\times 100$$

### Pre-formed biofilm disturbance assay

To determine the disturbance capacity of fractions, Meng *et al*’s 7 method was used. Accordingly, 20 *μl* of 1×10^6^ *CFU/ml* of culture was inoculated into 180 *μl* of MHB and incubated for 24 *hr* at 37 °*C*. Loosely bound cells were removed by PBS buffer. Fractions dissolved in DMSO (1 *mg/ml*) were added to the preformed biofilm in the wells and maintained DMSO alone was considered as the negative control while preformed biofilm alone as the positive control. Fractions dissolved in DMSO and medium alone were added to the clean wells and used as blank. The setup was incubated for 24 *hr* at 37 °*C* without shaking. Wells were washed thrice with PBS and stained to calculate the inhibition percentage using the above formula.

### GC-MS analysis of highly active column eluted fraction of R. patula

Chemical finger printing of highly active fraction was analyzed using JEOL GCMATE II GC-MS operated in EI mode at 70 eV (Agilent Technologies 6890N). In brief, 1 *μl* of fractions (0.1% *w/v*) was introduced into HP5 MS silica column (30 *m* × 0.25 *mm*). Injector temperature was 220*°C* and column temperature was fixed as 50–250*°C* at the rate of 10*°C min*^−1^. High pure helium was used as the carrier gas. Physical and chemical properties were determined by comparing the obtained spectra against spectral library of NIST EI-MS database which is the in-house retention index library of organic volatile compounds 23. Percentage of compounds was calculated using the formula:

$$\text{Percent}\;\text{compd}\;1=\frac{\text{Area}\;\text{of}\;\text{compd}\;1}{\text{Total}\;\text{srea}}\times 100$$

### In silico protein-ligand interaction studies

Compounds identified from the F44 of *R. patula* through GCMS chemical finger printing were drawn using Marvin sketch and stored as pdb files. The target protein DNA binding (LytTR) domain of AgrA was downloaded from PDB (PDB ID: 4G4K) and optimized by removing hetero-atoms and water molecules. Further side chains and missing atoms were added along with hydrogen to obtain a complete and refined structure using whatif server. DNA binding sites were identified through literature survey 24. AutoDockVina 25 was used to import all the selected compounds while Auto Dock 4.2 26 tool was used to check the affinity of ligand towards the active site of selected protein. Protein was prepared by adding Kollman charges and polar hydrogens. Ligand was prepared by adding Gasteiger charges and detecting root. Grid was generated by selecting active site residues (Ser231 to Ile238) where side chains of active site residues were flexible to interact with the ligand molecule (which is rotatable and stretches for interacting with neighboring ligand molecule). 50 long runs of docking were performed with Lamarckian Genetic Algorithm. However, different poses of interaction were observed in docking where the best fitting pose was selected for further visualization and analyzation of docked structure using Chimera software. Non bonded interactions were obtained using LigPlot+ v.1.4 27.

**Figure:**
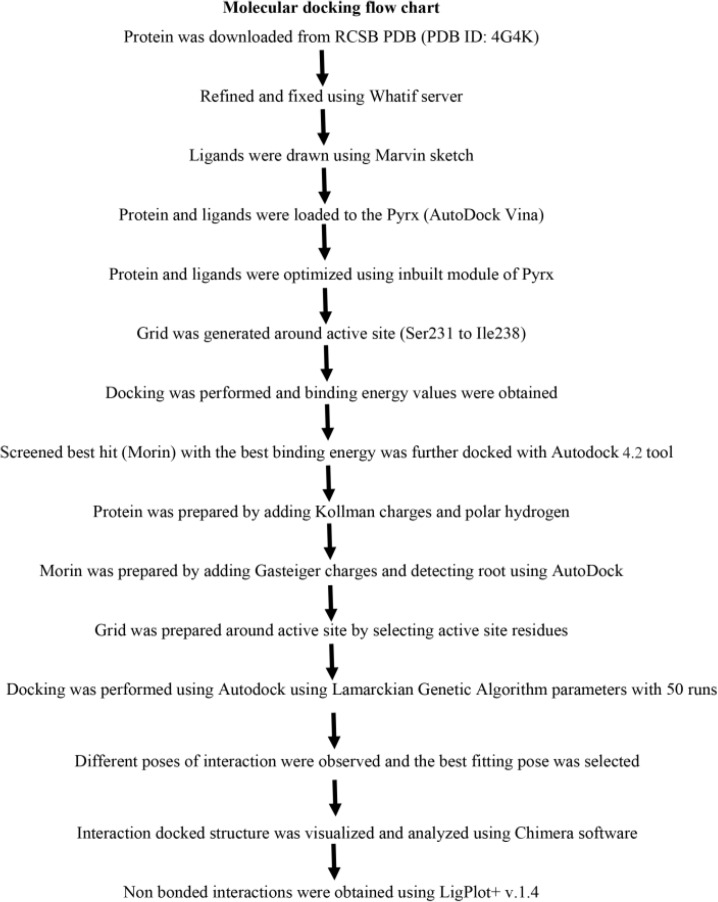


### Statistics analysis

All experiments were carried out in triplicates. Graph pad prism 6 was used to calculate mean and IC 50 value. T-test was performed to compare the mean values with the significance set at p<0.05.

## Results

### Antibacterial activity of fractions against S. aureus and MRSA

Disk diffusion method was performed to ascertain the antimicrobial effect of fractions against *S. aureus* and MRSA, which showed different degrees of antibacterial zones ranging from 0.5 to 1.5 *cm* (Table 1). Fraction F25 was highly active against MRSA, but no activity was observed in *S. aureus*. Unlike that, F53 was highly active against *S. aureus* while no activity was detected against MRSA. Interestingly, F44 was active against both *S. aureus* and MRSA. Generally, *S. aureus* is resistant to a number of antibiotics and produces enterotoxins 28. Presence of phytochemicals like fatty acids, phenols, flavonoids, alkaloids, terpenes and steroids reported in *R. patula* has proved their bioactive and antimicrobial activity 29. Fatty acids are considered as effective broad spectrum and non-specific antibiotics, which disrupt the oxidative phosphorylation and electron transport and also result in nutrient uptake impairment, thus inducing bacterial cell lysis 30. Therefore, different forms of fatty acids present in the extracts and polyphenolic compounds may play an important role as therapeutic agents and can attribute to antibacterial activity by different mechanisms like metabolism inhibition, membrane damage and inhibition of synthesis of cell wall, cell membrane and nucleic acid 2.

**Table 1.** Determination of antimicrobial and anti-quorum sensing ability of *R.patula* fractions eluted by Coloumn chromatography

**S. No**	**Fractions**	***S. aureus***			**MRSA**		

		**Zone of inhibition (*cm*)**	**Anti-adhesion (%)**	**Disturbance (%)**	**Zone of inhibition (*cm*)**	**Anti-adhesion (%)**	**Disturbance (%)**
1	F1	-	-	-	-	-	-
2	F2	-	-	-	-	-	-
3	F3	-	-	-	1.1	88.76	24.59
4	F4	-	-	-	-	-	-
5	F5	-	-	-	-	-	-
6	F6	-	-	-	-	-	-
7	F7	-	-	-	-	-	-
8	F8	-	-	-	-	-	-
9	F9	-	-	-	-	-	-
10	F10	-	-	-	-	-	-
11	F11	-	-	-		-	
12	F12	-	-	-	1	85.61	70.6
13	F13	-	-	-	1	79.14	84.44^##^
14	F14	-	-	-	-	-	-
15	F15	-	-	-	-	-	-
16	F16	-	-	-	-	-	-
17	F17	-	-	-	-	-	-
18	F18	-	-	-	1	-	-
19	F19	-	-	-	-	-	-
20	F20	-	-	-	-	-	-
21	F21	-	-	-	1	83.54	-
22	F22	-	-	-	-	-	-
23	F23	-	-	-	-	-	-
24	F24	-	-	-	-	-	-
25	F25	-	-	-	1.5^**^	82.29	67.58
26	F26	-	-	-	-	-	-
27	F27	-	-	-	-	-	-
28	F28	-	-		1.4	82.08	27.01
29	F29	-	-	-	-	-	-
30	F30	-	-	-	-	-	-
31	F31	-	-	-	-	-	-
32	F32	-	-		1	82.6	-
33	F33	-	-	-	-	-	-
34	F34	-	-	-	-	-	-
35	F35	-	-		1.3	86.46	75.82
36	F36	-	-	-	-	-	-
37	F37	-	-	-	-	-	-
38	F38	1.3	80.6	60.7	-	-	-
39	F39	-	-	-	1.4	89.17	59.41
40	F40	1.4	90.06^@^	77.2	-	-	-
41	F41	-	-	-	-	-	-
42	F42	-	-	-	-	-	-
43	F43	1.4	84.02	81.5^#^	-	-	-
44	F44^$^	1.2	89.9	51.6	1.4	86.8	72.1
45	F45	-	-	-	-	-	-
46	F46	-	-	-	-	-	-
47	F47	-	-	-	-	-	-
48	F48	-	-	-	-	-	-
49	F49	-	-	-	-	-	-
50	F50	1.3	77.2	66.88	-	-	-
51	F51	-	-	-	-	-	-
52	F52	-	-	-	-	-	-
53	F53	1.5*	64.8	48.93	-	-	-
54	F54	1.1	75.6	75.61	-	-	-
55	F55	-	-	-	1	95.2^@@^	61.06
56	F56	-	-	-	-	-	-
57	F57	-	-	-	-	-	-
58	F58	-	-		1	87.32	73.47
59	F59	-	-	-	-	-	-
60	F60	-	-	-	-	-	-

* Highest inhibition zone for *S. aureus.*

** Highest inhibition zone for MRSA.

@ Highest adhesion inhibition percentage of *S aureus.*

@@ Highest adhesion inhibition percentage of MRSA.

# Highest biofilm disturbance percentage of *S. aureus.*

## Highest biofilm disturbance percentage of MRSA.

$ Fraction having all the activity against both *S. aureus* and MRSA.

Terpenes like thymol, linalool and menthol have been reported to inhibit bacteria via membrane lipid bilayer penetration through fatty acyl chains and disrupting lipid packing thus altering cell membrane fluidity 23. Flavonoids have been reported as antimicrobial agents, which by inhibiting the nucleic acid synthesis damage cytoplasmic membrane and membrane functions by inhibiting the formation of D-alanine-Dalanine 2. Moreover, the antibacterial activity of fraction could be correlated with the amount of phenolic and flavonoid content present in the fraction and this perhaps may not be due to the single compound, but attributed to the combinations of interacting compounds 23,31. Thus, the antibacterial activity of the fractions could be due to the presence of bioactive compounds such as terpenes, phenols and flavonoids which can act as antibacterial agents by any of the above mechanism supporting the fact that plant fractions could be used as antibacterial agent.

### MIC of highly active fraction for respective organisms

**Figure 1.**
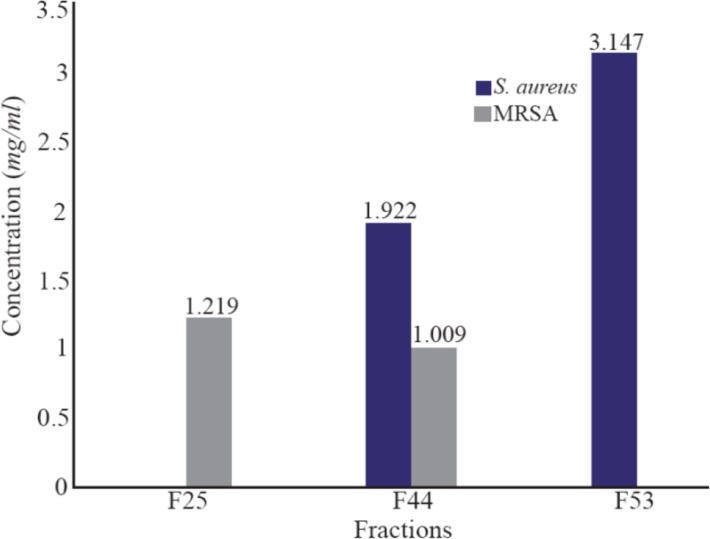
Determination of Minimum Inhibitory Concentration of highly active fractions of *R. patula.* The role of fractions indicate F44 to be active against both the selected strains of *S. aureus*.

MIC is the lowest concentration needed for complete inhibition of selected organism. In disk diffusion assay, the antibacterial activity depends on the diffusion nature of fractions and polarity of solvents used for fractionation which probably may contribute to higher activity at lower concentration or lower activity at higher concentration 32,33. Thus, to determine the IC50 value, highly active fractions (F25, F53 and F44) were further subjected to MIC determination against both *S. aureus* and MRSA using two fold broth dilution method. MIC value of F25 was determined as 1.219 *mg/ml* against MRSA while, 3.147 *mg/ml* was recorded for F53 against *S. aureus*. In case of F44, ranges from 0 to 78.27±1.48% were obtained against *S. aureus*, while 0 to 82.05±1.7% of inhibition was obtained for MRSA at respective concentrations. The results suggested F44 to be effective against both *S. aureus* and MRSA even at lower concentrations of 1.922 *mg/ml* and 1009 *μg/ml*, respectively (Figure 1). MRSA had lower IC50 value than *S. aureus* which shows that this fraction can inhibit MRSA effectively than *S. aureus*.

### Synergistic effect of highly active fractions with the combination of commercial drugs against selected organisms

Following the effect of antibacterial activity (against both MRSA and *S. aureus*) exhibited by the F44 (unlike F23 and F53, which acted against any one pathogen), all three were subjected to synergistic activity with commercial drugs such as chloramphenicol, vancomycin and tetracycline available as disks (each 30 *μg/disk*). Interestingly, the synergistic effect was not observed when the fractions were combined with chloramphenicol against both organisms. However, F53 alone when combined with tetracycline, synergistically reacted against *S. aureus*. The reason for tetracycline effectiveness and chloramphenicol failure may be due to the fact that, chloramphenicol as a broad spectrum bacteriostatic antibiotic, when combined with other antibiotics interfered with their action making them also inactive. This is perhaps evidenced by studies of Aqil *et al* 34, Beoni *et al* 35, Klastersky and Husson 36, and Jawetz *et al* 37 according to whom, synergistic interaction of chloramphenicol by the selected plant extracts and antibiotics against *S. aureus* actually failed, although it is a broad spectrum antibiotic which targets the cells protein synthesis but it still nullifies the effect by interfering with the other combined antibiotic.

**Figure 2.**
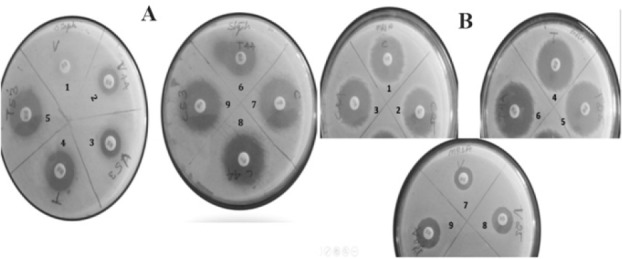
Determination Synergistic effect of highly active fractions with the combination of commercial drugs against *S. aureus* and MRSA. A) Synergistic effect of fractions on *S. aureus*; (1) Vancomycin alone; (2) Vancomycin with the combination of fraction F44; (3) Vancomycin with the combination of fraction F53; (4) Tetracycline alone; (5) Tetracycline with the combination of fraction F53; (6) Tetracycline with the combination of fraction F44; (7) Chloramphenicol alone; (8) Chloramphenicol with the combination of fraction F44; and (9) Chloramphenicol with the combination of fraction F44. B) Synergistic effect of fractions on MRSA; (1) Chloramphenicol alone; (2) Chloramphenicol with the combination of fraction F25; (3) Chloramphenicol with the combination of fraction F44; (4) Tetracycline alone; (5) Tetracycline with the combination of fraction F25; (6) Tetracycline with the combination of fraction F44; (7) Vancomycin alone; (8) Vancomycin with the combination of fraction F25; and (9) Vancomycin with the combination of fraction F44.

Thus, accordingly, in our study too, the selected organism treated with chloramphenicol and fractions simultaneously failed due to the interference of chloramphenicol on activity of our fractions. However, vancomycin showed synergistic effect with the combination of fractions, but when vancomycin alone was tested, there was no activity detected. Interesting results obtained from this test showed that the clinically isolated *S. aureus* was vancomycin resistant (Figures 2A and 2B) thus suggesting the fact that compounds present in the fractions with good activity could be identified as hit for Vancomycin-Resistant Staphylococcus Aureus (VRSA) infections along with MRSA infections. Hence, it necessitated the purification of phytochemicals present in this plant, which further could be explored to develop novel antibacterial compounds for the treatment of virulent and resistance strains of *Staphylococcus.*

### Time killing assay of highly active fraction F44 against S. aureus and MRSA

**Figure 3.**
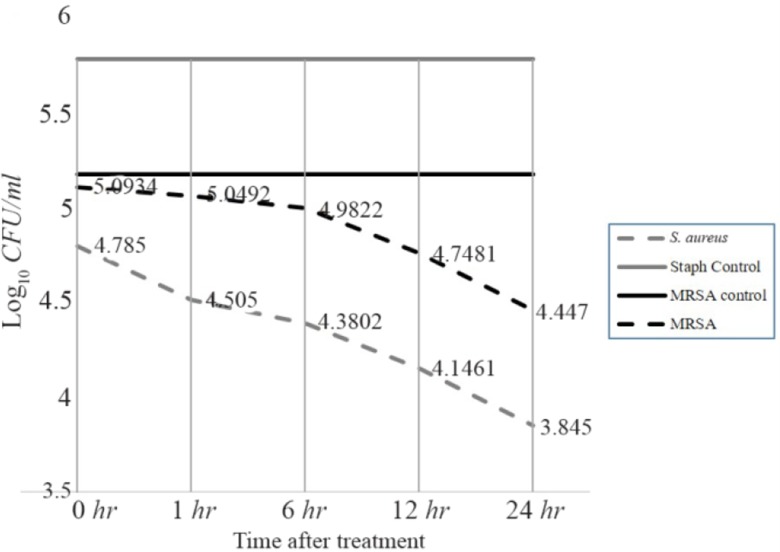
Time killing assay of fraction F44 against *S. aureus* and MRSA. The values represent the decrease in the growth to be directly proportional to the time.

Substances which kill or eliminate bacteria at the shortest time are considered bactericidal 29 whereas the ones that inhibit the bacterial growth are bacteriostatic 38. Accordingly, the shortest time taken by fraction F44 to kill or eliminate bacteria was determined by Time Killing assay (Figure 3), where MRSA colonies were reduced to 52.45% after one *hr* of contact to MIC concentration and further decreased to 39.34, 22.95 and 11.47% with increasing contact time of 6, 12 and 24 *hr*, respectively thus indicating the fact that the time exposed to fraction was directly proportional to the defense mediated by the fraction. However, this phenomenon was done to a greater extent (reduced to 90.32 in one *hr* and 77.41, 45.16 and 22.58% by the 6^th^, 12^th^ and 24^th^ *hr* respectively) in *S. aureus* indicating its suitability and favorability against those strains which are resistance to vancomycin, too (VRSA). Although based on the results, it could be observed that the selected fraction at determined concentration could not completely kill the organism at the given time (24 *hr*), it still had the ability to control the colony formation when compared to the control, suggesting the fact that fractions possess slow bactericidal activity against selected strains 39.

### Anti-biofilm Assay

The research focused on plant derived alternative medicine against bacteria was extended to control biofilm formation and was performed in two ways.

### Biofilm adhesion inhibition assay

Crystal violet assay was performed and different degrees of anti-adhesion effect by the fractions of *R. patula* were indicated to inhibit biofilm adhesion (Table 1). Twelve fractions showed activity towards MRSA (79.14–95.2%) and seven fractions had activity towards *S. aureus* (64.8–90.06%) revealing the effectiveness of fractions more against MRSA than *S. aureus*. However, highest inhibition percentage (95.2%) was obtained by F55 against MRSA and the lowest was obtained by F53 (64.8%) against *S. aureus* whereas in antibacterial activity F53 showed high inhibitory activity. Interestingly, F44 exhibited activity against both *S. aureus* (89.9%) and MRSA (86.8%) because as evidenced phytochemicals present in that fraction could have been responsible for inhibiting the biofilm adhesion by altering cell surface and or by inhibiting cell interactions to substance. Moreover, adhesion to the surface is the primary step to form biofilm and it depends on the characteristics of bacteria as influenced by the physicochemical properties of surface and environment 2. Thus, accordingly, different degrees of inhibition in our study could be revealed by the presence of flavonols of other phytoconstituents in the fractions attributed to anti-adhesion effect using different mechanisms.

### Disturbance assay of preformed biofilm

Disturbance effect of fractions on formed biofilm was evaluated which showed that fractions had different degrees of disturbance effect on *S. aureus* (48.93–81.5%) and MRSA (24.59–84.44%) (Table 1). Following the result of anti-adhesion assay, here too, F44 disturbed the biofilms of both *S. aureus* (51.6%) and MRSA (72.1%). It is difficult to control the formed biofilm ones and therefore they need to be inhibited at the time of biofilm initiation 23. Thus, the results of our study too consequently produced less disturbance than anti-adhesion. Although the mechanism of anti-biofilm is unknown, it is predicted that formed biofilm might be disturbed by the compounds present in the fractions by disturbing the process of adhesion through deactivating the enzymes responsible for surface attachment or cell-cell communication mediating signaling for cross-talks. Thus, accordingly, the formation of biofilm by *S. aureus* was inhibited by terpenes 23. Moreover, flavonoids such as apigenin, quercetin and myricetin were also reported to exhibit strong biofilm inhibition activity and exhibit enzyme modulation which is responsible for adherence of surface protein to the host cell and also responsible for virulence factors in surface protein 2. Hence, strong anti-biofilm activity in the present study could also be indicated by the adequate quantity of such compounds present in the fractions tested and therefore proceeded further to examine the compound identities.

### GC-MS analysis

**Figure 4.**
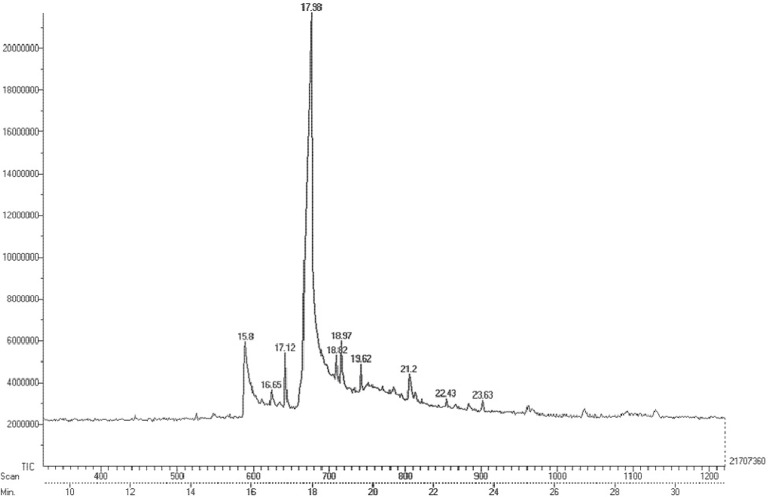
GC Chromatogram of highly active fraction F44. Finger print of the column eluted fraction44 demonstrates the peak area representing the separation of nine different compounds.

Fraction F44 was subjected to GC-MS for chemical finger printing analysis and nine compounds were identified (Figure 4). N-Hexadecanoic acid (Palmitic acid) was identified as the third compound and remarkably present in high concentration of 86.07% at RT 17.9 followed by Morin at RT 18.97 with 8.80%, while Oleic acid (RT 18.82), 4,8,12,16-Tetramethyl-heptadecan-4-olide (RT 21.2), 2-Cyclohexen-3,6-diol-1-one, 2-tetradecanoyl (RT 22.43) and Hexadecanoic acid, 2-bromo (23.63) were eluted with minimum concentra tion of below 1% (Table 2). Identification of these compounds in this fraction was helpful in understanding the mechanism of action of antibacterial and anti-biofilm activity. Anti MRSA activity achieved by flavanone is beneficial to the structure of A ring (5,7-dihydroxylation) and B ring (2′,4′- or 2′,6′-dihydroxylation), which is essential for significant activity 40. Thus, flavanone present in the fraction might be responsible for the antibacterial activity of MRSA. Genistein possesses exotoxin inhibition properties along with antibacterial activity 41, which might be one of the reasons for the potential activity of the fraction. Ohta *et al* 42 reported that palmitic acid had no antibacterial activity against *S. aureus* and MRSA, while Huang *et al* 43 reported that, long chain, medium chain and short chain fatty acids contribute to inhibition of biofilm and thereafter evolution and emergence of new species could be restricted. Thus, the presence of fatty acids might support biofilm activity of the fraction. Moreover, poly phenolic compounds possess anti-biofilm activity either by inhibition or by anti-adhesion. The poly phenolic compounds like flavone, 4’, 5,7-Trihydroxyisoflavone and Morin might be responsible for anti-biofilm activity of the fraction 44.

**Table 2.** Chemical finger prints of Fraction F44 by GC-MS

**S. No**	**RT**	**Chemical name**	**Molecular formula**	**Molecular weight**	**Peak area %**
**1**	15.8	Flavone	C_15_H_10_O_2_	222.239	2.492
**2**	17.12	4’,5,7-Trihydroxy isoflavone	C_15_H_10_O_5_	270.237	2.160
**3**	17.98	n-Hexadecanoic acid ^*^	C_16_H_32_O_2_	256.424	86.07
**4**	18.82	Oleic acid	C_18_H_34_O_2_	282.461	0.058
**5**	18.97	Morin	C_15_H_10_O_7_	302.236	8.809
**6**	19.62	Z-5,17-Octadecadien-1-ol acetate	C_20_H_36_O_2_	308.499	0.129
**7**	21.2	4,8,12,16-Tetramethylheptadecan-4-olide	C_21_H_40_O_2_	324.541	0.160
**8**	22.43	2-Cyclohexen-3,6-diol-1-one, 2-tetradecanoyl	C_20_H_34_O_4_	338.488	0.044
**9**	23.63	Hexadecanoic acid, 2-bromo	C_16_H_31_BrO_2_	335.326	0.070

* Compound present in highest concentration.

### Molecular docking studies

*AgrA* gene plays a significant role in quorum sensing and survival of *S. aureus.* Nine different compounds (Table 2) identified through GC-MS for the fraction F44 were drawn through Marvin sketch and those files were subjected to AutodockVina interaction software, which accepts multiple compounds to understand their interaction ability on the single target protein. Thus, accordingly, nine different compounds subjected at the active site (Ser231 to Ile238) on the DNA binding domain of agrA protein, revealed that all compounds interact with the protein as evidenced through the calculated binding energies, which ranged from −3.6 *kcal/mol* to −7.1 *kcal/mol*. However, in comparison with the study of Leonard *et al* 24, the apo form of the protein interacted with the tested compounds and exhibited the binding energies between −3.5 *kcal/mol* to −4.1 *kcal/mol* for different compounds such as 4-phenoxyphenol, 9 H-xanthene-9-carboxylic acid, 2-(4-methylphenyl)-1,3-thiazole-4-carboxylic acid, *etc*.

## Discussion

**Figure 5.**
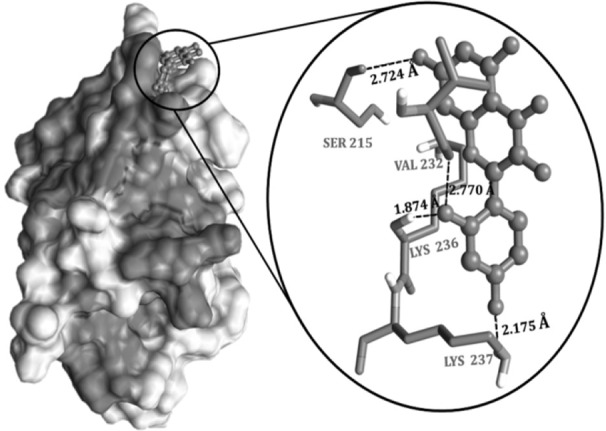
Docking interaction of Morin with DNA binding (LytTR) domain of AgrA. The interaction pattern highlights the efficiency of morin inhibiting the interactive residues at the target site.

Thus, based on the above comparison, the compounds under investigation showed better occupancy; however, Morin (C_15_H_10_O_7_) exhibited good interaction pattern with les-ser binding energy of −7.1 *kcal/mol*, followed by genistein with −6.8 *kcal/mol* and flavone with −6 *kcal/mol* energy, respectively (Table 3). Therefore, Morin was proceeded to have a better insight of molecular interactions using AutoDock 4 tool. The Grid box was generated considering the conserved regions between Ser231 to Ile238 which form a shallow groove for the DNA binding site and exhibit 100% identity among 211 *Staphylococcal* strains of Uniprot database 24. Morin interacted with the DNA binding region where it formed four hydrogen bonds (Table 4 and Figure 5). Both Val232-O and Lys236-NH exhibited hydrogen bond with O_2_’ of hydroxyphenyl moiety, while Lys237-H interacted through O_4_’ of hydroxylphenyl moiety and Ser215 bonded with Chromen-4-one moiety of Morin, respectively. Additionally, the interactions were stabilized by electrostatic (with Lys236 and Arg233) and hydrophobic interactions (with Val-232 and Val235) with the domain region of agrA. Thus, from the interaction pattern, it is clear that the highly active residues, which are also considered as key residues involved in regulating the DNA binding activity at the active pocket, were actually engaged in bonding with our ligand-Morin, proving its ability to inhibit the activity of agrA and therefore mimic the DNA, which thereby may suppress the upregulation of virulence factor genes. However, the better binding energies compared to Leonard PG *et al* 24, may also be attributed to another important catalytic active polar residue SER215, which formed hydrogen bond through O7 of Chromen-4-one moiety of Morin, as this was not reported in earlier studies of Leonord *et al* 24 that may have influenced the formation of binding energies and could be responsible for the stronger interaction as evidenced through the bond lengths (Table 4). Hence, considering the fact that interaction of Morin at binding site could quench the quorum sensing ability and therefore disturb the cell wall synthesis and destruct the growth of the organism (unaccompanied data), if proven through wet lab studies, it may be applied for all 211 strains of *Staphylococcus* and other pathogens possessing LytTR domain in agrA proteins.

**Table 3.** Determination of binding energies of identified compounds with DNA binding (LytTR) domain of agrA using Auto Dock vina

**S. No**	**Ligand**	**Binding energy (*kcal/mol*)**
**1**	Flavone	−6
**2**	4’,5,7-Trihydroxy_isoflavone	−6.8
**3**	n-Hexadecanoicacid	−3.6
**4**	Oleic acid	−3.8
**5**	Morin^*^	−7.1
**6**	Z-5,17-Octadecadien-1-ol_acetate	−4.1
**7**	4,8,12,16-Tetramethylheptadecan-4-olide	−4
**8**	2-Cyclohexen-3,6-diol-1-one, 2-tetradecanoyl	−4.3
**9**	Hexadecanoicacid, 2-bromo	−4.1

* Protein –compound complex, which had best binding energy.

**Table 4.** Hydrogen bond interactions of morin with DNA binding (LytTR) domain of AgrA

**S. No**	**Residues**	**Atom of residues**	**Ligand atoms**	**Distance (*Å*)**
**1**	LYS237	H_2_	O_4’_	2.175
**2**	SER215	O	O_7_	2.724
**3**	VAL232	O	O_2’_	2.770
**4**	LYS236	NH	O_2’_	1.874

## Conclusion

The work uncovered the importance of fractions of *R. patula* by means of anti-bacterial and anti-biofilm activity, thereby supported the medicinal use of this plant in siddha medicine. Fraction F44 was positive for all activities against both VRSA and MRSA. Chemical finger printing of F44 added valuable information to the anti-bacterial activity of that fraction. Hence, the phytochemicals present in *R. patula* can be used as anti-biotic against both VRSA and MRSA. Molecular docking of Morin with DNA binding domain of agrA revealed it as the potential agent for quorum sensing thereby enabling it to be used against all staphylococcus bacteria including MRSA and other pathogens. However, further research on the purification of this compound will enable to elucidate the mechanism of natural process of Morin on biofilm inhibition, which is also basically suggested as the futuristic study.
